# MicroRNA-101 negatively regulates Ezh2 and its expression is modulated by androgen receptor and HIF-1α/HIF-1β

**DOI:** 10.1186/1476-4598-9-108

**Published:** 2010-05-17

**Authors:** Paul Cao, Zhiyong Deng, Meimei Wan, Weiwei Huang, Scott D Cramer, Jianfeng Xu, Ming Lei, Guangchao Sui

**Affiliations:** 1 Department of Cancer Biology and Comprehensive Cancer Center, Wake Forest University School of Medicine, Winston-Salem, NC 27157, USA; 2 Center for Cancer Genomics, Wake Forest University School of Medicine, Winston-Salem, NC 27157, USA; 3 College of Life Science, Shaanxi Key Laboratory for Molecule Biology of Agriculture, Northwest A&F University, Yangling, 712100 Shaanxi Province, PR China

## Abstract

**Background:**

In prostate cancer (PCa), the common treatment involving androgen ablation alleviates the disease temporarily, but results in the recurrence of highly aggressive and androgen-independent metastatic cancer. Therefore, more effective therapeutic approaches are needed. It is known that aberrant epigenetics contributes to prostate malignancy. Unlike genetic changes, these epigenetic alterations are reversible, which makes them attractive targets in PCa therapy to impede cancer progression. As a histone methyltransferease, Ezh2 plays an essential role in epigenetic regulation. Since Ezh2 is overexpressed and acts as an oncogene in PCa, it has been proposed as a bona fide target of PCa therapy. MicroRNAs (miRNAs) regulate gene expression through modulating protein translation. Recently, the contribution of miRNAs in cancer development is increasingly appreciated. In this report, we present our study showing that microRNA-101 (miR-101) inhibits Ezh2 expression and differentially regulates prostate cancer cells. In addition, the expression of miR-101 alters upon androgen treatment and HIF-1α/HIF-1β induction.

**Result:**

In our reporter assays, both miR-101 and miR-26a inhibit the expression of a reporter construct containing the 3'-UTR of Ezh2. When ectopically expressed in PC-3, DU145 and LNCaP cells, miR-101 inhibits endogenous Ezh2 expression in all three cell lines, while miR-26a only decreases Ezh2 in DU145. Ectopic miR-101 reduces the invasion ability of PC-3 cells, while restored Ezh2 expression rescues the invasiveness of PC-3 cells. Similarly, miR-101 also inhibits cell invasion and migration of DU145 and LNCaP cells, respectively. Interestingly, ectopic miR-101 exhibits differential effects on the proliferation of PC-3, DU-145 and LNCaP cells and also causes morphological changes of LNCaP cells. In addition, the expression of miR-101 is regulated by androgen receptor and HIF-1α/HIF-1β. While HIF-1α/HIF-1β induced by deferoxamine mesylate (DFO) decreases miR-101 levels, the overall effects of R-1881 on miR-101 expression are stimulatory.

**Conclusions:**

This study indicates that miR-101 targets Ezh2 and decreases the invasiveness of PCa cells, suggesting that miR-101 introduction is a potential therapeutic strategy to combat PCa. MiR-101 differentially regulates prostate cell proliferation. Meanwhile, the expression of miR-101 is also modulated at different physiological conditions, such as androgen stimulation and HIF-1α/HIF-1β induction.

## Background

Enhancer of zeste homolog 2 (Ezh2) is a member of the polycomb group (PcG) protein family involved in suppressing gene expression through remodeling chromatin [1]. As a histone methyltransferase, Ezh2 catalyzes histone H3 lysine 27 (H3-K27) trimethylation [2], which is a hallmark of gene silencing [3]. Ezh2 is an important component of the polycomb repressive complex 2 (PRC2) and is required in maintaining gene silencing. The association of Ezh2 with other PcG proteins is essential to its methyltransferase function, since the pharmacologic disruption of PRC2 inhibits the methylation of H3-K27 [4]. Ezh2 needs to be recruited by DNA binding proteins, such as YY1 and E2F, to associate with chromatin and exert its function [5,6]. In addition, Ezh2 and its associated PcG proteins regulate various biological processes, including X-chromosome inactivation [7], stem cell self-renewal and exhaustion [8,9], skeletal muscle differentiation [10], actin polymerization [11] and circadian clock function [12].

Increasing evidence suggests an essential role of Ezh2 in cancers. Numerous studies indicate that Ezh2 overexpression is a common phenomenon in prostate cancer (PCa) that is associated with a poor clinical outcome of PCa patients [6,13-15]. Therefore, Ezh2 was proposed to be a bona fide oncogene [13] and its increase can be used as a marker of prostate tumors with aggressive and metastatic potential. Several studies also suggested the prospect of Ezh2 as a therapeutic target in PCa treatment. Ezh2 knockdown by small interfering RNA (siRNA) decreases prostate cell proliferation [13] and inhibits the metastatic tumor growth of PC-3 cells in bone tissue [16]. On the other hand, Ezh2 promotes proliferation and invasion of PCa cells [17] and ectopically expressed Ezh2 in prostate cells enhances proliferation [18]. These studies indicate a role of Ezh2 in aggressive PCa and suggest that Ezh2 may be a therapeutic target of PCa treatment [18]. Taken together, Ezh2 plays an oncogenic role in PCa and elucidating the mechanisms that regulate Ezh2 function may provide fundamental therapeutic insight in treating this cancer.

Previous studies demonstrate Ezh2 can be regulated at the transcriptional or translational level. The tumor suppressor p53 [19] and transcription factor E2F [6] can bind to the promoter of the Ezh2 gene to inhibit or transactivate its expression, respectively. In addition, Ezh2 undergoes post-translational modification by Akt, which decreases its methyltransferase activity [20]. Recently, increasing evidence indicates microRNAs (miRNAs) can regulate gene expression at the post-transcriptional level. Therefore, we wanted to study whether Ezh2 is also regulated by miRNAs.

MicroRNAs are a group of small RNAs with 17-24 nucleotides that regulate gene expression through interfering mRNA translation [21]. Ample evidence indicates that miRNAs may regulate tumorigenesis by functioning as either oncogenes or tumor suppressors [22]. Interestingly, different cancers exhibit characteristic miRNA signatures in miRNA expression profiling studies [23]. However, gaps still exist in understanding the precise mechanisms of miRNA-mediated cancer development and progression. Due to the importance of Ezh2 in PCa progression and its potential as a therapeutic target in PCa therapy, identifying miRNA(s) that regulates Ezh2 expression may lead to the development of novel therapeutic approaches in PCa treatment. A recent study indicated that the genomic loss of miR-101 leads to Ezh2 overexpression in human cancer samples, suggesting the physiological significance of miR-101-regulated Ezh2 function in PCa development [24]. In the current study, we demonstrate that miR-101 negatively regulates Ezh2 expression in PCa cells and miR-101 expression is affected by androgen stimulation and HIF-1α/HIF-1β induction.

## Materials and methods

### Antibodies and Reagents

Antibodies against Ezh2 (4905), HIF-1β/ARNT (3718S), Tri-Methyl-Histone H3-Lys 27 (H3K27m3, 9756), and Lamin A/C (2032) were purchased from Cell Signaling Technology (Danvers, MA). HIF-1α antibody was kindly provided by Dr. Constantinos Koumenis (University of Pennsylvania School of Medicine). Histone H3 (C-16, sc-8654) and Androgen Receptor antibodies (N-20, sc-816) were purchased from Santa Cruz Biotechnology (Santa Cruz, CA).

### MicroRNAs, siRNAs, DNA plasmids and transfection

All microRNA mimics (miR-101, miR-26a and a scramble control) were synthesized by Dharmacon, Inc. (Chicago, IL), with the following sequences: miR-101, UACAGUACUGUGAUAACUGAA; miR-26a: UUCAAGUAAUCCAGGAUAGGCU; and scramble control (miR-cont): UCACAACCUCCUAGAAAGAGUAGA. The siRNAs for control (GGG CCA TGG CAC GTA CGG CAA G) and Ezh2 (GGT GAT CAC AGG ATA GGT ATT) were delivered by a lentiviral vector, pLU carrying anti-puromycin cDNA [25].

To generate a reporter construct, we amplified a 493-base pair (bp) DNA fragment consisting of the last 50 bps of Ezh2 coding region and 443 bps of the 3'-UTR of Ezh2 mRNA from human genomic DNA (P/N: 5-0109, Affymetrix, Inc.). This 3'-UTR region of Ezh2 containing the predicted target sites of miR-101 and miR-26a was then subcloned downstream of Gaussia luciferase (GLuc) that is driven by a phosphoglycerate kinase (PGK) promoter. We also constructed plasmids with mutated target sites of these miRNAs. The recognition of the seed sequence of a miRNA to the 7-8 nucleotides at the 3' end of its target site is essential to miRNA-mediated translational inhibition. Thus, in the predicted target sites of miR-101 and miR-26a in Ezh2 3'-UTR, we replaced these nucleotides recognized by the seed sequences of these two miRNAs with scrambled sequences, respectively. As a result, we generated three control reporter plasmids with the two miR-101 target sites mutated, either individually or combinatorially (^45^AGTACTGT^66 ^to A**CCG**C**G**G**C**, and/or ^101^GTACTGTA^121 ^to **C**T**G**C**A**G**AT**, mutated nucleotides are in bold), and one control reporter plasmid with the mutated miR-26a target site (^236^TACTTGAA^257 ^to **CTGCA**G**CT**). To express Ezh2 in PC-3 cells, Ezh2 cDNAs (generously provided by Dr. Sartorelli [10]) were individually subcloned into a Lentiviral vector, pSL4, which co-expresses the puromycin N-acetyltransferase to render infected cells resistant to puromycin.

### Cell culture, transient transfection and Lentiviral production

PWR-1E, LNCaP, DU145 and PC-3 cell lines were obtained from the American Type Culture Collection (Manassas, VA). LNCaP, DU145 and PC-3 cells were maintained in RPMI 1640 supplemented with 1% penicillin/streptomycin and 10% fetal bovine serum. In androgen starvation experiments, LNCaP cells were also cultured in phenol red-free RPMI media containing 1% charcoal-stripped FBS (Invitrogen), and treated with 0, 0.01, 0.1, and 10 nM R1881 (a synthetic non-aromatizable androgen). PC-3 cells were also treated for 0 and 6 h by 100 μM deferoxamine mesylate (DFO, Sigma), dissolved in RPMI culturing media. PWR-1E cells were maintained in keratinocyte-SFM (Invitrogen). Lipofectamine 2000 (Invitrogen) was used in transient transfection of PC-3 cells based on the protocol provided by the manufacturer. SiPort NeoFX lipofectamine (Ambion) was used in transient transfection of LNCaP based on the manufacturer's protocol. The production of Lentivirus followed a previously reported protocol [26]. Briefly, 293T cells were transfected with either an empty pSL4 vector, pSL4-Ezh2, pLU-cont (control) siRNA, or pLU-Ezh2 siRNA, together with three packaging plasmids (pMDLg/pRRE, pRSV-RSE and pVSV-G) using a transfection protocol of calcium phosphate-DNA precipitation. Medium containing viral particles was collected 48 h after transfection. Lentiviruses in the medium were concentrated by ultracentrifugation at 25,000 rpm, 4°C for 90 min and stored at -80°C. To infect cells, concentrated Lentivirus was added to the cells with the medium containing 8 μg/ml polybrene. The medium was replaced with normal culture medium 6 h post viral addition.

### Luciferase reporter assay

Each well of PC-3 cells cultured on a 12-well plate was transfected with 50 ng of a reporter or mutated reporter plasmid, miR-101 or miR-26a mimic (with a final concentration of 100 nM in each well), 100 ng of plasmid expressing secreted alkaline phosphatase (SEAP) driven by a β-actin promoter and other expression plasmids if needed. Aliquots of medium from the transfected wells were collected 48 h posttransfection to measure luciferase activity. Fifty μl of the medium (diluted if necessary) was mixed with 100 μl of substrate solution containing 0.5 μg/ml of coelenterazine (CTZ), 200 mM NaCl, 50 mM Tris·HCl and 0.01% Triton X-100, at pH 8.7. The light emission was measured at a wavelength of 480 nm and normalized with the SEAP expression [27].

### Histone extraction

Cells were resuspended in Triton Extraction Buffer (phosphate buffered saline, containing 0.5% Triton X-100, 2 mM phenylmethylsulfonyl fluoride, 0.02% NaN_3_), lysed on ice with gentle shaking for 10 min, and centrifuged at 2,000 rpm for 10 min in 4°C. The cell pellet was washed with the Triton Extraction Buffer and centrifuged as above. To extract the histones, the cell pellet was resuspended in 0.2 N HCl and incubated overnight at 4°C with gentle shaking. After centrifuging at 2,000 rpm for 10 min in 4°C, the supernatant containing histone extract was transferred to a new tube and analyzed by Western blot.

### Clonogenic assay

PC-3 cells were transfected with control miRNA (miR-cont) or miR-101 mimics using Lipofectamine 2000 (Invitrogen). Forty-eight hours post-transfection, cells were plated at various densities (125, 250, 500, 1000 and 2000) in 6-cm cell culture dishes. After 7-10 days, the cells were fixed with 10% formalin and stained with 0.1% crystal violet. Colonies with 50-cells or greater were counted on each dish.

### Androgen Starvation and Bicalutamide treatment

LNCaP cells were seeded and cultured in complete medium for 48 h. The medium was replaced by phenol red-free RPMI media containing 1% charcoal-stripped FBS (Invitrogen). Cells were allowed to adapt to this androgen deprivation condition for an additional 24 h before treating with 0, 0.01, 0.1, 1.0, and 10 nM R1881. For the treatment of inhibiting androgen receptor, LNCaP cells in the androgen deprivation condition were pre-treated with 5 μM of bicalutamide (B9061, Sigma) before R1881 addition.

### WST-1 cell proliferation assay

LNCaP, DU-145 and PC-3 cells were assayed differently. LNCaP cells were plated at a density of 6000 cells/well in 96-well plates and transfected with the control miRNA (miR-cont) or miR-101 mimics using siPort NeoFX lipofectamine (Ambion). At each time point, cell proliferation in triplicates was measured using WST-1 (Roche) following the manufacturer's protocol. DU-145 and PC-3 cells were transfected with miR-cont or miR-101 mimics using Lipofectamine 2000 (Invitrogen) in 6-well plates. Forty-eight hours post-transfection, cells were seeded in triplicate at a density of 2000 cells/ml in 96-well plates. Cell proliferation at each time point was determined as described above.

### Real-Time RT-PCR analysis

Total RNA from cells was extracted using TRIzol reagent (Invitrogen). Levels of mature forms of miR-101 or miR-26a in cells were determined by TaqMan MicroRNA Assays (Applied Biosystems), and data were normalized to U6 expression (Applied Biosystems). To determine the levels of Ezh2 mRNA in cells, 2 μg of RNA was incubated with 0.5 μg/μl of oligo dT primer (Promega) at 70°C for 5 min. The following reverse transcriptase mix was then added and incubated at 42°C for 1 h: 5 μl of 5× MMLV buffer, 5 μl of 10 mM dNTP, 0.6 μl of RNasin, 1 μl of MMLV reverse transcriptase, 13.4 μl of nuclease-free water. Quantitative PCR analysis using Taqman Gene Expression Assays was then performed for Ezh2 expression, and data were normalized to GAPDH expression (Applied Biosystems). All analyses were performed using the ABI7000 sequence detection system. The ΔΔC_T _method [28] was used to calculate relative expression.

### Matrigel invasion assay

One hundred μl of Matrigel (BD Biosciences, diluted to 1 mg/ml in serum free-cold cell culture media) was added into an upper chamber of a 24-well transwell plate and incubated at 37°C for 4 h until the Matrigel solidified. Cells to be tested were starved in FBS-free medium for 18-24 h, then harvested by trypsinization and washed 3 times with medium containing 1% FBS and resuspended in the same medium at a density of 1 × 10^6 ^cells/ml. The polymerized Matrigel was gently washed with warmed serum-free culture media. One hundred μl of the cell suspension was added on top of the Matrigel, while 650 μl of complete medium with 10% FBS was added to each bottom chamber. The assembled chamber was incubated at 37°C in a cell culture incubator for 48 h. The medium in the top and bottom chambers was carefully aspirated, followed by washes with PBS. The cell invasion was quantified by counting the cells stained by crystal violet.

### Migration Assay

LNCaP cells were transfected by miR-cont or miR-101 mimics. After 72 h, the cells were resuspended in RPMI with 1% FBS at a density of 1 × 10^5 ^cells/ml. Five hundred μl of the cell suspension was added to the upper chamber (Becton Dickinson, 35-3097, 8 μm pore size), while 750 μl of RPMI with 5% FBS was added to the lower chamber. The chambers were incubated at 37°C in a cell culture incubator for 24 h. Cell migration was quantified by counting the cells stained by crystal violet.

### Wound healing assay

LNCaP cells transfected by miR-cont or miR-101 mimics were seeded in a 6 well plate and cultured for 72 h to obtain 80% monolayer confluency. A wound was created by scraping the cells using a plastic pipette tip, and the medium was replaced with fresh medium. Images were captured immediately (day 0) and every day for 5 days. Cell migration was qualitatively assessed by the size of the wounds at the end of the experiment.

## Results

### The 3'-UTR of Ezh2 mRNA contains conserved target sites of miR-101 and miR-26a

**Figure 1:**
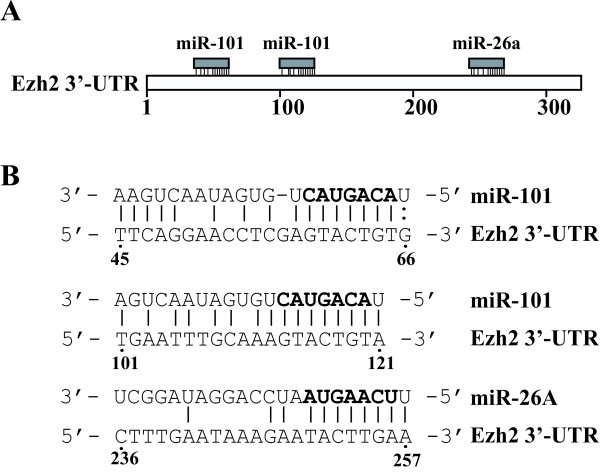
**Schematic diagrams of predicted targets of miR-101 and miR-26a in Ezh2 3'-UTR**. **A. **Two predicted miR-101 target sites and one predicted miR-26a target site in the 3'-UTR of Ezh2. The first nucleotide right behind the stop codon of Ezh2 is arbitrarily designated as "1". **B. **Sequence alignments of miR-101 and miR-26a with their corresponding potential target sites in the 3'-UTR of Ezh2. The seed sequences of the two miRNAs are bolded, and the matched or complementary nucleotides between the miRNAs and the Ezh2 3'-UTR are indicated. The positions of each predicted target on Ezh2 3'-UTR are labeled beneath the alignment.

To identify miRNAs that potentially regulate Ezh2 expression, we analyzed the 3'-UTR sequence of human Ezh2 using an algorithm available from the Sanger Institute (see [29] and http://microrna.sanger.ac.uk/) to predict potential miRNA target sites. We identified a number of miRNA candidates that may regulate Ezh2 expression. We chose miR-101 and miR-26a for further study, because these two miRNAs showed high binding scores and their target sites on the 3'-UTR of Ezh2 are conserved in a wide range of species, including human, monkey, mouse, chicken and platypus. In addition, when we used other algorithms of miRNA target prediction [30-32] to analyze the Ezh2 sequence, these two miRNAs also appeared in the lists as top candidates. Importantly, two recent studies indicated that miR-26a targets Ezh2 [33,34] and other studies suggested these two miRNAs are downregulated in cancers [35,36]. If we arbitrarily designate the nucleotide right after the Ezh2 mRNA stop codon as "1" and its downstream side as "+", two predicted targets sites of miR-101 are present at 45-66 and 101-121, while one miR-26a target site is at 236-257 (Fig. 1A). The alignment of miR-101 and miR-26a with their putative target sequences on the human Ezh2 3'-UTR is depicted in Fig. 1B.

### Expression of miR-101 and miR-26a in prostate cancer cell lines

**Figure 2:**
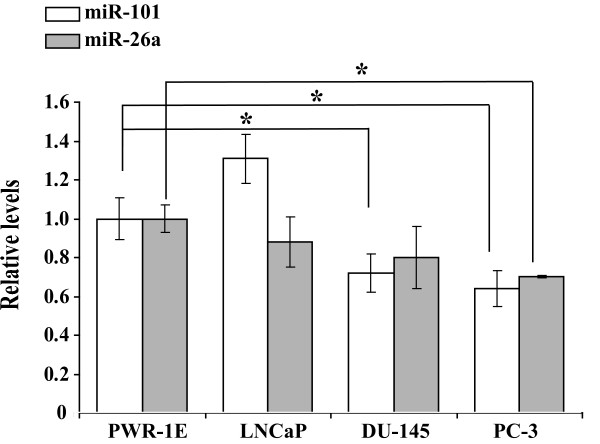
**Analysis of miR-101 and miR-26a expression in prostate cancer cell lines**. RT-PCR was conducted using RNAs extracted individually from PWR-1E, LNCaP, DU-145 and PC-3 cells with a stem-loop primer specific to miR-101 or miR-26a. The generated cDNAs were subjected to further analysis by Taqman Real-Time PCR using FAM-labeled miR-101 or miR-26a probes (Applied BioSystems). Each sample was analyzed in triplicates. The data are presented as an average of two experiments and normalized to the expression of the endogenous U6 RNA using the ΔΔC_T _method [28] as described in Materials and Methods. Student T-test was used to determine statistical significance and the asterisks indicate that the p values are not higher than 0.05.

Previous studies on miRNA profiles revealed decreased levels of miR-101 and miR-26a in PCa [35,36]. To determine the correlation between the expression of these two miRNAs and the malignancy of PCa cell lines, we used Real-Time RT-PCR to study the expression of mature form miR-101 and miR-26a in four different prostate cell lines: PWR-1E, LNCaP, DU-145 and PC-3. While PWR-1E is a non-tumorigenic prostate epithelial cell line [37], the other three cell lines are tumorigenic and exhibited increasing aggressiveness in an order of LNCaP, DU-145 and PC-3, as described in a previous study [38]. As shown in Fig. 2, DU-145 and PC-3 cells showed markedly decreased expression of miR-101, when compared to PWR-1E cells (34% and 41% decrease, respectively, p < 0.05), while LNCaP cells exhibited a slight increase of miR-101 expression (Fig. 2). Although the inverse correlation between miR-26a expression and the aggressiveness of these four cell lines was only marginal, the difference in the expression between PC-3 and PWR-1E was still significant (30% decrease, p = 0.05).

### Effects of ectopically expressed miR-101 and miR-26a on Ezh2 expression

**Figure 3:**
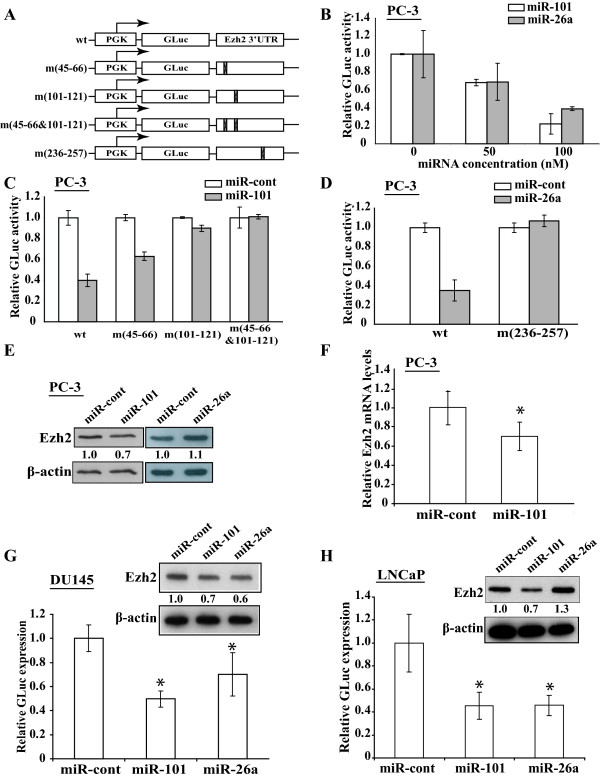
**Analysis of miR-101 and miR-26a on Ezh2 3'-UTR in reporter assay, and endogenous Ezh2 expression**. **A. **In wild type (wt) reporter, a 493-bp fragment containing the last 50 bps of Ezh2 coding region and the first 443 bps of Ezh2 3'-UTR embracing the predicted miR-101 and miR-26a target sites is subcloned downstream of GLuc driven by PGK promoter. The four reporter constructs contain mutations in Ezh2 3'-UTR at miR-101 target sites: m(45-66), m(101-121) and m(45-66&101-121); and at miR-26a target site: m(236-257). **B. **Increasing miR-101 and miR-26a (0, 50 and 100 nM, compensated with miR-cont to 100 nM, if necessary) were cotransfected with wt reporter presented in **"A" **and the SEAP-expressing plasmid into PC-3 cells (triplicated). GLuc activity was determined and normalized by SEAP activity (see Materials and Methods for details). **C. **One hundred nM of miR-cont or miR-101 was cotransfected with 50 ng of the indicated reporter constructs and the SEAP-expressing plasmid. GLuc activity was measured and normalized as described above. **D. **The experiment was performed as **"C" **using miR-26a and reporter plasmids as labeled. **E. **PC-3 cells were transfected with miR-cont, miR-101, or miR-26a (120 nM). Ezh2 and β-actin expression was determined by Western blot. Relative Ezh2 levels normalized by β-actin are indicated. **F. **Ezh2 mRNA levels (normalized to GAPDH) in microRNA-transfected cells analyzed by Real-Time RT-PCR. The asterisk: p ≤ 0.05. **G **and **H**. GLuc activity measurement (triplicated) and Western blot were performed as **C, D **and **E **in DU-145 (**G**) and LNCaP (**H**) cells.

To determine whether miR-101 and miR-26a target the 3'-UTR of Ezh2, we studied the effects of these two miRNAs on the reporters containing GLuc and the Ezh2 3'-UTR. The intact (wt) reporter and its mutated versions with replaced nucleotides at the binding sites of miR-101 or miR-26a are shown in Fig. 3A. When increasing amounts (0, 50, 100 nM) of miR-101 and miR-26a were transfected into PC-3 cells, the reporter construct with intact Ezh2 3'-UTR showed decreased GLuc expression (Fig. 3B). After we mutated the two miR-101 target sites simultaneously, the generated reporter construct, m(45-66&101-121), completely lost the response to the transfected miR-101 (Fig. 3C). However, when we mutated the two target sites individually, we observed that the GLuc expression of the two reporter constructs, m(45-66) and m(101-121), could be partially repressed by miR-101 (Fig. 3C). Interestingly, mutations in nucleotide (nt) 101-121 of Ezh2 3'-UTR led to a more profound effect than that of nt 45-66, suggesting miR-101 interacts with these two sites differentially. Similarly, a reporter construct with mutated miR-26a target site, m(236-257), also lost the inhibition to the reporter construct (Fig. 3D). The reporter assay studies thus suggest that the presence of these miRNA target sites in Ezh2 3'-UTR of the reporter construct is necessary for the inhibition by miR-101 and miR-26a.

To determine the regulation of miR-101 and miR-26a on endogenous Ezh2 expression, we individually transfected the synthetic mimics of miR-101 and miR-26a in PC-3 cells and studied the Ezh2 expression by Western blot. As shown in Fig. 3E, ectopically expressed miR-101 decreased the expression of endogenous Ezh2 protein, suggesting that miR-101 negatively regulates Ezh2 mRNA translation. Interestingly, miR-26a did not show any detectable inhibition to Ezh2 (Fig. 3E), although it efficiently repressed the expression of the reporter construct containing Ezh2 3'-UTR (Fig. 3B). In addition to the protein changes, we also detected a significant decrease of Ezh2 mRNA in PC-3 cells transfected by miR-101 compared to the cells transfected by miR-cont (30% ± 15, p < 0.05, Fig. 3F).

We also extended these studies in other prostate cell lines. Reporter assays in DU145 and LNCaP cells showed that miR-101 and miR-26a, but not miR-cont, significantly inhibited the expression of the reporter construct with Ezh2 3'-UTR. In DU145 cells, both miR-101 and miR-26a decreased the expression of Ezh2 (Fig. 3G). However, in LNCaP cells, miR-101, but not miR-26a, downregulated Ezh2 expression (Fig. 3H).

Previous studies indicated that mutations or polymorphism in the 3'-UTR of a gene could abolish its responsiveness to the regulation of miRNAs [39,40]. Therefore, we asked whether the inertness of the endogenous Ezh2 expression to ectopic miR-26a was due to the alteration of the miR-26a target site at Ezh2 3'-UTR in PC-3 cells. We amplified the region in the genomic DNA of PC-3 cells containing the miR-26a target site and analyzed the PCR fragment by DNA sequencing. However, we did not find any change in this predicted miR-26 target site when compared with the human genomic DNA sequence in NCBI (data not shown).

### Effects of ectopic miR-101 on the proliferation, survivability and invasiveness of prostate cancer cells

**Figure 4:**
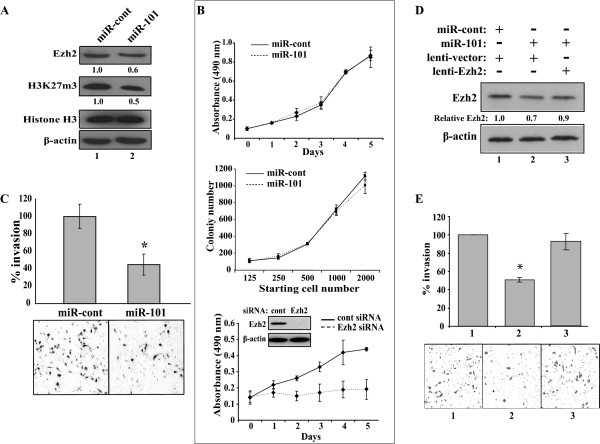
**Effects of ectopically expressed miR-101 on the growth, survivability and invasiveness of PC-3 cells**. **A. **Effect of ectopic miR-101 on histone H3-K27 methylation. PC-3 cells were transfected with miR-cont or miR-101 (120 nM). Aliquots of transfected cells were analyzed by Western blots using the indicated antibodies. Relative protein expression is indicated at the bottom of each image. **B. **Effects of Ezh2 downregulation on proliferation and colony formation of PC-3 cells. In the top and middle panels, aliquots of transfected PC-3 cells in **"A" **were studied by WST-1 proliferation assay (top) and clonogenic assay (middle). In the bottom panel, PC-3 cells infected by lentivirus carrying the control and Ezh2 siRNAs were tested by WST-1 assay and Ezh2 knockdown tested by Western blot was indicated. **C. **Effects of ectopic miR-101 on the invasiveness of PC-3 cells using aliquots of transfected PC-3 cells in **A**. The asterisk indicates p < 0.05 and representative images are presented. **D **and **E**. Effects of restored Ezh2 expression on miR-101 transfected PC-3 cells. In **D**, PC-3 cells were either infected with lentivirus generated from pSL4 vector or pSL4-Ezh2 as indicated. At 48 h post infection, cells were transfected with 120 nM of miR-cont or miR-101 as labeled. After another 48 h, cells were analyzed by Western blots using the indicated antibodies. In **E**, aliquots of the infected/transfected PC-3 cells with the corresponding sample numbers (1, 2 and 3) in **D **were studied by invasion assay. Percent invasion is shown with "*" indicating p < 0.05 and representative images.

Since our data indicate that miR-101 inhibited Ezh2 expression, we asked whether the miR-101-mediated Ezh2 decrease may affect the histone methylation and the PC-3 cell growth, survivability and invasiveness. Ezh2 regulates the expression of its target genes through mediating histone H3-K27 tri-methylation [41]. Therefore, we examined this modification in PC-3 cells transfected by miR-101. As shown in Fig. 4A, ectopic expression of miR-101 led to the concomitant decrease of both Ezh2 and histone H3-K27 methylation, while the total histone H3 remained unchanged. Using these cells to determine the effect of miR-101 on cell growth, we did not detect significant change in cell proliferation (Fig. 4B, top panel). Meanwhile, the clonogenic assay showed that miR-101 did not change the colony formation of PC-3 cells, which suggested unchanged cell survivability (Fig. 4B, middle panel). We asked whether the modest Ezh2 decrease by miR-101 in PC-3 cells is insufficient to cause any change in cell growth. Therefore, we infected PC-3 cells by lentivirus carrying Ezh2 siRNA that could knock down the endogenous Ezh2 by over 90%. In the cell proliferation study, these Ezh2-siRNA transduced PC-3 cells exhibited marked defects in cell proliferation compared to the cells expressing a control siRNA (Fig. 4B, bottom panel).

We further studied the invasive capability of these cells using the Matrigel invasion assay. As shown in Fig. 4C, the PC-3 cells expressing ectopic miR-101 exhibited significantly decreased ability of penetration (46% ± 12, p < 0.05) compared to the cells expressing miR-cont, suggesting that ectopic miR-101 inhibited the invasiveness of PCa cells. Since the correlation between Ezh2 inhibition and attenuated PCa progression has been well-documented [13,17], we asked whether the Ezh2 decrease by miR-101 is the primary cause of the compromised invasiveness of miR-101 transfected cells. We infected the miR-101 transfected PC-3 cells with lentivirus expressing Ezh2, which could restore the Ezh2 expression to a comparable level (90%) of the endogenous Ezh2 (Fig. 4D). In the invasion assay, the PC-3 cells with restored Ezh2 expression exhibited similar penetration ability to the miR-cont transfected cells (92% ± 2.6, p < 0.05, compare columns 3 and 1 of Fig. 4E), suggesting that miR-101 attenuates the invasiveness of PC-3 cells primarily through downregulating Ezh2 expression.

**Figure 5:**
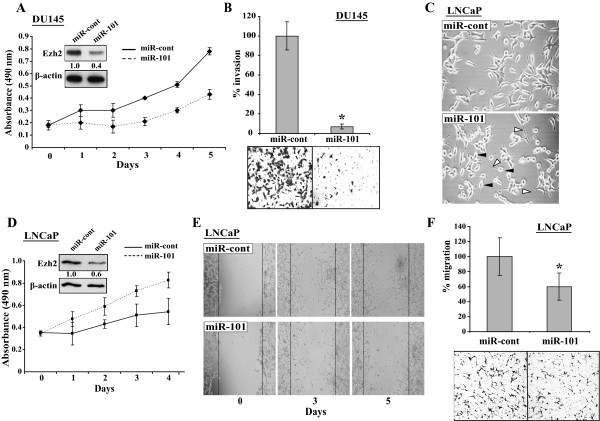
**Effects of miR-101 on DU-145 and LNCaP cells**. **A and B. **Effects of ectopic miR-101 on the proliferation and invasiveness of DU-145 cells. DU-145 cells were transfected by 120 nM of miR-cont or miR-101 mimics and cells were collected at 72 h post-transfection. Aliquots of the cells of each treatment were studied in triplicates by (**A**) WST-1 cell proliferation assay and (**B**) Matrigel invasion assay. Western blot analyses of these transfected cells are shown as an insert of "**A**" and representative images of invasion assay are also shown in "**B**" (right panel). **C**, and **D**. Effects of ectopic miR-101 on the morphology and proliferation of LNCaP cells. In **C**, the LNCaP cells at 72 h post transfection were imaged under microscope (20×). The black arrow heads indicate the rounding of the cell body, while the white arrow heads point the extensions of cell cytoplasmic portion. In **D**, the LNCaP cells were seeded in triplicate at a density of 6000 cells/well in 96-well plates and transfected with 120 nM of miR-cont or miR-101 mimics. Cell proliferation was studied by WST-1 reagent. **E**. Wound healing assay of LNCaP cells transfected with miRNA mimics (120 nM). Images were captured at the time points as indicated. **F**. Boyden chamber migration assay of LNCaP cells transfected with miRNA mimics (120 nM). The cell numbers were quantified after crystal violet staining with represented images shown below.

We asked if the phenomenon of PC-3 cells with ectopically expressed miR-101 could be extended to other prostate cell lines. Therefore, we studied the effects of ectopic miR-101 on DU-145 and LNCaP cells. In DU-145 cells, we observed that miR-101 inhibited both cell proliferation and invasion (Fig. 5A and 5B). This result is consistent with a recent study demonstrated by Chinnaiyan group [24]. Both PC-3 and DU-145 are aggressive and androgen receptor (AR) negative PCa cell lines. We further studied the effects of miR-101 on LNCaP cells that have relatively low aggressiveness and are AR positive. We first observed that LNCaP cells expressing ectopic miR-101 exhibited a morphological change with extension of the cytoplasmic portion and rounding of the cell body, compared to the typical fusiform morphology of LNCaP cells expressing the miR-cont (Fig. 5C). This might not be caused by neuroendocrine differentiation, since several markers for neuroendocrine cells did not show significant changes (data not shown). It is noteworthy that these morphological changes were not observed in PC-3 and DU-145 cells transfected by miR-101 (data not shown). Unexpectedly, LNCaP cells expressing ectopic miR-101 displayed increased cell proliferation compared to miR-cont transfected cells, which is opposite to the effects of miR-101 on DU-145 cells (Fig. 5D). This phenomenon is reproducible in multiple independent experiments. Since these morphological changes of LNCaP cells may alter their cytoskeletal structure and affect cell migration, we assessed the migration of these cells using wound healing and Boyden Chamber cell migration assays. In both studies, LNCaP cells transfected by miR-101 exhibited decreased migrating rates than the cells with miR-cont (Fig. 5E and 5F).

### Effects of HIF-1α/HIF-1β and androgen receptor on the expression of miR-101

**Figure 6:**
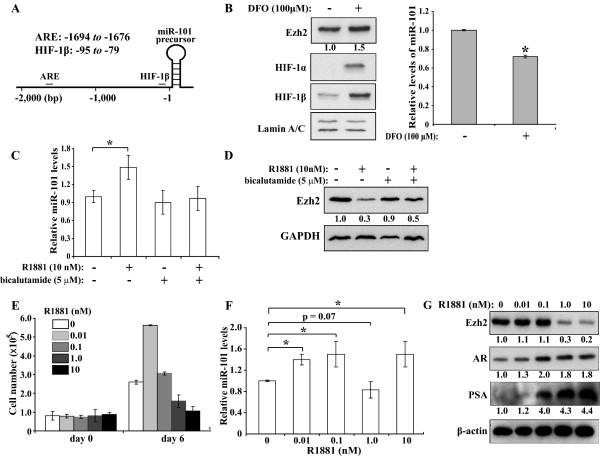
**Effects of HIF-1β and AR on miR-101 expression**. **A. **Schematic diagram of HIF-1β and AR binding elements upstream of miR-101 coding region. **B. **Protein and miR-101 expression of DFO-treated PC-3 cells. Nuclear proteins of PC-3 cells treated with 100 μM DFO or mock were analyzed by Western blot (left panel) and Real-Time RT-PCR (right panel, average of two individual experiments with triplicated samples). **C **and **D**. LNCaP cells were treated by R1881 and bicalutamide as indicated and analyzed by (**C**) Real-Time RT-PCR for miR-101 (average of three individual experiments) and (**D**) Western blot for Ezh2. **E, F **and **G. **Effects of R1881 on cell growth, miR-101 levels and gene expression in LNCaP cells. In **E**, cells were seeded at 1 × 10^5 ^cells/well on 6-well plates in phenol red-free RPMI medium with 1% charcoal-stripped FBS, followed by treatment of 0, 0.01, 0.1, 1.0, and 10 nM of R1881. At days 0 and 6, cells in triplicates were counted. Data are the averages of three or more individual experiments. In **F**, miR-101 levels in the extracted RNA at day 6 were determined by Real-Time RT-PCR with each sample analyzed in triplicate. Data are the average of four independent experiments. Student t-test was used to determine statistical significance (* indicates p < 0.05). In **G**, whole cell lysates at day 6 of the treatment were analyzed by Western blot using the antibodies labeled on the left.

Like protein-coding mRNAs, miRNAs are also transcribed from their genes in genomic DNA. Therefore, the expression of each miRNA is driven by a promoter and regulated by transcription factors [42]. To investigate the mechanism regulating miR-101 expression, we analyzed the upstream region of its coding sequence in the human genome using an algorithm that predicts transcription factor binding elements on DNA [43]. We identified a number of proteins that have the potential to regulate miR-101 expression and focused on the regulatory proteins with both high binding probability scores and previously reported roles in cancers. Two of these proteins are hypoxia inducible factor-1β (HIF-1β) and AR, whose binding elements are located upstream of the miR-101 precursor at -95 to-79 and -1694 to -1676, respectively (Fig. 6A).

HIF-1β interacts with HIF-1α to form a heterodimer that regulates the transcription of hypoxia-responsive genes in PCa angiogenesis and progression [44]. HIF-1α expression can be stimulated by various signaling pathways. Therefore, to determine the effect of HIF-1β on miR-101, we treated PC-3 cells with 100 μM of deferoxamine mesylate (DFO), an iron chelator that induces HIF-1α expression [45]. As shown in the left panel of Fig. 6B, at 6 h post DFO treatment, PC-3 cells exhibited an increase of Ezh2 expression with concomitant increases of both HIF-1α and HIF-1β compared to the mock-treated cells. Importantly, the miR-101 levels in the DFO-treated PC-3 cells exhibited a significant decrease to 72% (p < 0.05) compared to the control (right panel, Fig. 6B), suggesting that the HIF-1α/HIF-1β heterodimer downregulates miR-101.

To test how AR regulates miR-101 expression, we treated androgen dependent LNCaP cells with 10 nM of R1881 and observed a significant increase of miR-101 (50% ± 20, Fig. 6C). To determine if this phenomenon was due to the AR's function, we pretreated LNCaP cells with 5 μM of bicalutamide, an antagonist of AR. As a result of inhibiting the functional AR, we abolished the miR-101 induction caused by R1881 (Fig. 6C). Consistently, with the induction of miR-101 by R1881, we observed concurrent Ezh2 decrease, which was partially restored by bicalutamide treatment (Fig. 6D).

AR plays an essential role in the development of normal prostate gland and the growth of PCa [46]. R1881 can both induce the differentiation and affect the proliferation of LNCaP cells [47,48]. Especially, different concentrations of R1881 exert differential effects on the growth of LNCaP cells [47]. Therefore, we studied miR-101 and Ezh2 expression of LNCaP cells cultured in medium with 1% charcoal-stripped serum and 0, 0.01, 0.1, 1 or 10 nM of R1881. Cells with these treatments were counted at days 0 and 6 to evaluate their growth, while the miR-101 levels and protein expression were determined by Real-Time PCR and Western blot, respectively. As shown in Fig. 6E, LNCaP cells exhibited biphasic growth rates in response to R1881. While 0.01 nM of R1881 stimulated the growth of LNCaP cells, further increases of R1881 adversely affected the cell proliferation. This observation is consistent with a previous study of R1881's effects on LNCaP cells [47]. Meanwhile, the treatments of 0.01, 0.1 and 10 nM of R1881 significantly increased miR-101 expression in LNCaP cells (by 40% ± 14, 50% ± 29 and 50% ± 26, respectively. p < 0.05, n = 4, Fig. 6F). However, for unknown reasons, 1 nM of R1881 did not significantly change miR-101 levels (p = 0.07, n = 4), which was reproducible in 4 individual studies. The effect of R1881 was validated by the marked PSA induction in response to increasing R1881 concentrations, consistent with previous studies [47,48]. Meanwhile AR levels were also elevated (Fig. 6G). In addition, the overall Ezh2 expression negatively correlated to the AR increases and miR-101 induction, except at the 1.0 nM of R1881 (Fig. 6F and 6G).

## Discussion

Prostate tumorigenesis is accompanied by deregulated gene expression. It has been well established that aberrant DNA methylation and histone modifications contribute to these processes. Recently, changes of miRNA profile in cancer cells and their roles in tumorigenesis have been increasingly appreciated. Since the major biological function of miRNAs is mediating gene expression, we investigated how essential genes in prostate tumorigenesis are regulated by different miRNAs, and whether the miRNA-mediated gene expression is important in PCa development. Multiple studies indicated that Ezh2 is potentially a prognostic marker and therapeutic target of PCa [15,18,49]. Therefore, our initial study investigated whether Ezh2 expression is regulated by miRNAs in prostate tumorigenesis.

In this report, we demonstrated that the 3'-UTR of Ezh2 contains the target sites of both miR-101 and miR-26a and these sites are conserved among different species. However, while ectopic expression of miR-101 decreased the endogenous Ezh2 in all three tested cell lines, miR-26a only inhibited Ezh2 in DU-145 cells, but not in PC-3 and LNCaP cells. The mechanism underlying this observation is still unclear and advanced understanding of the dynamic regulation of miRNAs may provide an explanation of this phenomenon. Currently, several algorithms are available to predict potential miRNA target sites in a given mRNA sequence. Most of them employ a scoring system that identifies highly complementary sites using dynamic programming alignment and rewards complementarity at the 5' end of the microRNA. However, these algorithms cannot predict whether a potential target site is blocked by a secondary structure or RNA binding protein(s), which makes it inaccessible [50]. A recent report also indicates that single nucleotide polymorphisms inside microRNA target sites affect miRNA-mediated gene inhibition and consequently influence tumor susceptibility [40]. Therefore, the presence of a miRNA target site in a mRNA does not assure that the gene is regulated by this miRNA. On the other hand, a handful of evidence suggests that genes regulated by certain miRNAs may not contain canonical target sites predicted by most algorithms [51-53]. Since our DNA sequencing analysis did not detect any mutation at the potential miR-26a target site in Ezh2 3'-UTR of PC-3 cells, we predict that lack of inhibition of miR-26a to the endogenous Ezh2 expression may be due to the interference of RNA binding proteins in PC-3 and LNCaP cells. When the 493-bp DNA fragment containing 443-bp of Ezh2 3'-UTR was subcloned into the reporter construct, these potential RNA binding proteins that interfere with the miRNA binding may have been saturated due to the robust expression of the reporter plasmid. This may explain the responsiveness of the reporter construct to the ectopic miR-26a in all three cell lines. Since the composition of the cellular proteins and microenvironment can be cell type specific, the response of endogenous Ezh2 expression to miR-26a may also change. Therefore, miR-26a could downregulate Ezh2 in DU145 cells, but not in PC-3 and LNCaP cells (Fig. 3G). In addition, miR-26a was also reported to negatively regulate Ezh2 expression in myoblasts and lymphoma cells [33,34].

MicroRNAs have been implicated in fine tuning the expression of target genes to physiologically relevant levels [54,55]. Therefore, consistent with previous reports on miRNAs' modulation of other gene expression, ectopically expressed miR-101 did not dramatically dampen the expression of endogenous Ezh2, although it exhibited much more pronounced inhibition to the luciferase reporter construct. Certainly, miR-101 must not be the only regulator of Ezh2 expression and it very likely collaborates with other miRNAs or transcription factors to adjust Ezh2 expression under different physiological conditions. However, it is reasonable to predict that downregulated miR-101 contributes to the increased expression of Ezh2 observed in PCa cells relative to the levels seen in normal prostate cells.

We observed ectopic miR-101 inhibited Ezh2 expression and led to markedly decreased invasiveness of all three tested cell lines. Importantly, our results of the negative regulation of Ezh2 by miR-101 are consistent with a recent report from the Chinnaiyan group showing that the genomic loss of miR-101 causes Ezh2 upregulation in PCa [24]. At least in PC-3 cells, this phenomenon was due to the concomitantly reduced Ezh2 expression, since ectopically expressed Ezh2 restored the invasiveness (Fig. 4E). Consistently, we also observed the inhibitory effects of miR-101 on the invasiveness of DU145 cells and migrating ability of LNCaP cells (Fig. 5B and 5F). However, miR-101 exhibited differential effects on the proliferation of PC-3, DU-145 and LNCaP cells (Fig. 4B, 5A and 5D). While ectopic miR-101 caused growth defects of DU-145 cells and did not affect that of PC-3 cells, it surprisingly promoted LNCaP cell proliferation with concurrent morphological changes that were not observed in PC-3 and DU-145 cells. These results indicate that the ectopic miR-101 has differential effects in different prostate cell lines. The mechanisms underlying these phenotypic discrepancies need further investigation. It is also noteworthy that PC-3 cells are more aggressive than DU-145 and LNCaP cells, and PC-3 and DU145 are both androgen independent and AR negative. LNCaP cells are androgen dependent and have the lowest malignancy among these three cell lines. Thus, the effects of miR-101 introduction on cell proliferation may rely on the aggressiveness and AR status of the cells.

LNCaP cells with ectopic miR-101 exhibited a morphological change. In a time-lapse video microscopy study, we observed that these cells formed cytoplasmic extensions, reminiscent of filopodia, which was not shown in the cells expressing miR-cont (data not shown). When we further investigated whether this morphological change affected cell migration, we detected the decreased migrating rates in miR-101 transfected LNCaP cells compared to the cells with miR-cont (Fig. 5E-F). It is unclear whether Ezh2 regulates these alterations. We predict that other miR-101 regulated genes involved in cell migration may play a role in this morphological change of LNCaP cells.

We did not detect any significant difference of Ezh2 mRNA levels among PWR-1E, LNCaP, DU-145 and PC-3 cell lines, which is consistent with a previous study [13]. These results suggest that at least in these cell lines Ezh2 overexpression in PCa is likely regulated at posttranscriptional levels, but not at transcription.

The effects of HIF-1β and AR on miR-101 indicate that the expression of miRNAs respond to the physiological and environmental changes. The DFO-induced expression of HIF-1α and HIF-1β led to decreased miR-101 expression, suggesting that miR-101 is a component in hypoxia-regulated signaling pathways. We also observed the upregulation of miR-101 in LNCaP cells treated by 10 nM of R1881, which was attenuated by an AR antagonist, bicalutamide. It is noteworthy that bicalutamide alone did not significantly decrease miR-101 levels (Fig. 6C), suggesting that without androgen stimulation AR does not have any detectable effect on miR-101 expression. However, in our further studies, although R1881 at 0.01 and 0.1 nM showed similar effects to that at 10 nM, the 1 nM of R1881 did not show any stimulation to miR-101 expression in multiple experiments (Fig. 6F). Meanwhile, at 0.01 nM of R1881 with increased miR-101 expression, cell proliferation was unexpectedly enhanced (Fig. 6E and 6F). The mechanisms to interpret these unexpected changes are unclear. We predict that differential activation of androgen-stimulated signaling pathways may contribute to them. Especially at 1 nM of R1881, certain proteins or pathways that need to be identified may have been altered, which causes the bypass of AR's regulation to miR-101 and consequently leads to the observation inconsistent with these at other R1881 levels. Multiple previous studies also demonstrated the differential effects of androgen on prostate cell proliferation [47,48].

We also noticed that at certain concentrations (0.1 and 1.0 nM) of R1881, the expression levels of miR-101 and Ezh2 (Fig. 6F and 6G) did not show a correlation as we observed in Fig. 6C. We predict that other R1881/androgen receptor-regulated pathways may play a role in mediating Ezh2 expression at these conditions.

Overall, the regulation of HIF-1α/HIF-1β and androgen receptor suggests a role of miR-101 in tumor progression and normal prostate development. Further investigation is needed to delineate the mechanisms underlying the altered expression of miR-101 during prostate cancer hypoxia and prostate cell differentiation.

## Conclusions

Current therapeutic treatment of PCa frequently leads to reoccurred cancers with more aggressive and refractory characteristics. Hence, it is crucial to identify new therapeutic targets and develop more effective approaches to treat this disease. Ezh2 regulates histone methylation and contributes to the aberrant epigenetics in PCa. Importantly, Ezh2 overexpression correlates with the pathological degrees and tumor progression of this cancer, suggesting its potential as a therapeutic target. MiR-101 negatively regulates Ezh2 expression and concurrently attenuates the invasion ability of prostate cancer cells, which can be rescued by ectopically expressed Ezh2. This implicates that the inhibition of Ezh2 by miR-101 is a prospective approach to be used as a new strategy in PCa therapy. Moreover, the levels of miR-101 fluctuate upon the androgen treatment and HIF-1α/HIF-1β induction, suggesting it is differentially regulated at different physiological conditions. Overall, our study indicates that miR-101 plays a regulatory role in prostate tumorigenesis and restoring miR-101 levels may be an effective approach for PCa treatment.

## Competing interests

The authors declare that they have no competing interests.

## Authors' contributions

PC and GS initiated the project and designed the study. PC performed the experiments. MW and WH assisted with the Matrigel Invasion Assay and some other experiments. ZD, SDC, JX and ML participated in the design of the study. All authors helped in discussing, reading, and proofreading the final manuscript.
